# Anomalous Origin of the Left Pulmonary Artery: Hemi-Truncus Arteriosus

**DOI:** 10.21699/ajcr.v8i2.556

**Published:** 2017-03-18

**Authors:** Ali Shabbir Hussain, Mariam Shakir, Shabina Ariff, Rehan Ali, Babar Hassan

**Affiliations:** The Aga Khan University Hospital Karachi, Pakistan

**Keywords:** Pulmonary artery, Anomaly, Hemi-truncus arteriosus

## Abstract

Hemi-truncus arteriosus is a rare congenital cardiovascular malformation. It usually presents in infancy and leads to development of progressive pulmonary vascular disease, heart failure, and death. We report a case of hemi truncus arteriosus in a 12-day-old neonate who was successfully managed at our institute.

## INTRODUCTION

Hemi-truncus arteriosus refers to the anomalous origin of pulmonary artery from aorta. It was first described by Fraentzel in 1868 as a rare form of truncus arteriosus with the incidence of 0.1% of all congenital cardiac anomalies. Most of the patients with hemi-truncus arteriosus are diagnosed in infancy.[1,2] In most of the patients right pulmonary artery abnormally originates from the posterior aspect of the ascending aorta near the aortic valve. Less commonly, the left pulmonary artery abnormally originates from the ascending aorta and is usually associated with a right aortic arch. Blood flow from the right side of the heart goes to the lung via normally originating pulmonary artery whereas additional blood from the abnormally originating pulmonary artery (hemi-truncus) carrying oxygenated blood from aorta to the lungs causes volume and pressure overload to the lungs leading to progressive pulmonary vascular disease, heart failure and eventually death.[3,4] We are reporting an interesting case of the same anomaly treated successfully by timely diagnosis and management.

## CASE REPORT

A 12-day-old female baby was received with the complaints of reluctance to feed and episodes of respiratory distress since birth. Baby was delivered to a primigravida mother (no antenatal follow-ups) at term via emergency lower segment cesarian section due non-progress of labor with good APGAR score. Baby developed tachypnea, respiratory distress and issues of desaturations within 24 hours of life therefore shifted from well-baby center to NICU of our hospital. The preliminary workup was done in well-baby center. Antibiotics and oxygen support was given. On arrival, the baby was tachypneic, otherwise vitally stable and maintaining oxygen saturation of 98% on 2 liters flow. She was non-dysmorphic and anthropometric measures were normal with a weight of 2.5 kilograms. Systemic examination and baseline laboratory workup were in normal range. X-ray chest showed bilateral perihilar infiltrates. Cardiac shadow appears to be magnified in supine position. Echocardiography was done on 16th day of life, which showed small patent foramen ovale (PFO) with additional fenestration, elevated left atrial pressures with strong suspicion of left hemi-truncus, moderate right ventricular hypertension, moderate right ventricular dilation with mild to moderate hypertrophy, qualitatively mild to moderate right ventricular systolic dysfunction and normal left ventricular systolic function. A computerized tomographic angiogram showed anomalous origin of the left pulmonary artery from the arch of aorta (right sided) as its first branch, appearance was consistent with hemi-truncus arteriosus along with normal origin of the right pulmonary artery which was a continuation of the pulmonary trunk. The main pulmonary artery was continuing as the right pulmonary artery and measures 8mm in diameter, causing increase pulmonary blood flow (Fig. 1, 2).

After establishing the diagnosis, baby was managed with minimal oxygen and low dose of diuretics to prevent increase fluid over-load and referred to cardiothoracic surgeon where corrective surgery was performed at one month of age. Left pulmonary artery (LPA) was dissected from ascending aorta, ligated and divided. LPA was translocated to main pulmonary artery and very small anterior wall was augmented with pericardium. Echocardiography was repeated on 1st post-operative day which revealed normal dominant left ventricle with good functions. Baby was eventually discharged from hospital and is doing well, gaining weight and being regularly followed by our neonatology and cardiology team.

**Figure:**
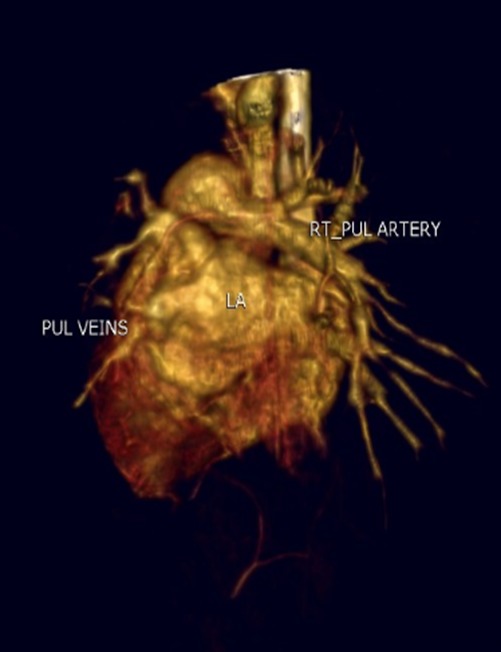
Figure 1. C.T angiogram images(3-D reconstruction) showing Anomalous origin of the left pulmonary artery from the arch of aorta as its first branch.

**Figure:**
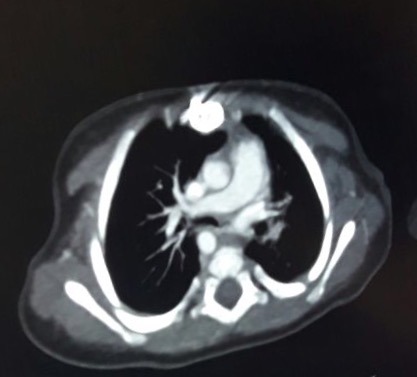
Figure 2: Post contrast axial image showing abnormal origin of left pulmonary artery from arch of aorta.

## DISCUSSION

Hemi truncus arteriosus is a rare cardiac structural anomaly. Children who are diagnosed with this anomaly early and undergo early surgical repair, have relatively better outcomes. Infants who do not undergo surgical correction have a 70% first-year mortality rate, and 30% of the infants die within 3 months. Early repair is carried out to avoid pulmonary vascular disease of the lung.[4] The repair should be contemplated within the first six months of life to prevent severe pulmonary vasculature obstructive disease. In our case, we referred the baby earlier and corrective surgery was performed on time, which led to favorable outcome in our case.

The anomalous origin of the left pulmonary artery, in contrast, is thought to result from failure of development of the left sixth arch and persistence of the left fifth arch.(5) If left untreated, the pulmonary bed is vulnerable to early onset of pulmonary vascular obstructive disease owing to the large blood supply to both lungs. It is important to recognize this rare congenital cardiac anomaly early and contemplate surgical repair for better prognosis.

## Footnotes

**Source of Support:** Nil

**Conflict of Interest:** None declared

